# Clinicopathological profile and outcomes of anorectal melanoma from a tertiary care center in India

**DOI:** 10.2144/fsoa-2021-0091

**Published:** 2022-02-10

**Authors:** Vikas Garg, Sameer Rastogi, Harshal Aswar, Shamim A Shamim, Ekta Dhamija, Adarsh Barwad, Rambha Pandey, Rajesh Panwar, Ashish Upadhyay

**Affiliations:** 1 Department of Medical Oncology, BRAIRCH, AIIMS, Delhi, 110029 India; 2 Department of Nuclear Medicine, AIIMS, Delhi, 110029 India; 3 Department of Radiodiagnosis, BRAIRCH, AIIMS, Delhi, 110029 India; 4 Department of Pathology, AIIMS, Delhi, 110029 India; 5 Department of Radiation Oncology, BRAIRCH, AIIMS, Delhi, 110029 India; 6 Department of GI Surgery & Liver Transplantation, AIIMS, Delhi, 110029 India; 7 Department of Biostatistics, AIIMS, Delhi, 110029 India

**Keywords:** anal, anorectal, chemotherapy, immunotherapy, melanoma, mucosal, rectal

## Abstract

**Background:**

Anorectal melanoma (AM) is a rare subtype of melanoma.

**Aim:**

To study the clinic–pathologic features and outcomes in patients with AM.

**Materials & methods:**

Clinical, pathologic findings and outcomes of patients with AM were recorded.

**Results:**

Twenty-seven patients with AM were identified with median age of 57 years. Most patients presented in stage III (44.4%). Lymph node involvement was seen in 70.4%. The response to chemotherapy and immunotherapy was 16.6 and 25.0%, respectively. At a median follow up of 11 months, median overall survival was 30 months. Ballantine stage 3 and weight loss at presentation were predictors of poor survival.

**Conclusion:**

AM presents at an advanced stage with lymph node and distant metastasis.

## Plain language summary

Anorectal melanoma is a rare subset of melanoma presenting at an advanced stage with lymph node and distant metastasis. Early disease is managed surgically; however, optimal treatment of advanced disease is unclear. The stage at diagnosis is the most important prognostic factor. Prognosis is poor, but with the advent of targeted therapy and immunotherapy, the outcomes are improving.

Mucosal melanomas are exceedingly rare and constitute around 1.3% of all melanomas. Most common site of mucosal melanomas is head and neck (55.4%), followed by anorectal (23.8%), vulvovaginal regions (18%) and urinary tract (2.8%). These are more common in females, have greater proportion in non-white population as compared with cutaneous melanoma (CM) and have advanced age (median 70 years) at the time of presentation [1,2].

Anorectal melanomas (AM) constitute just 0.05% of all colorectal malignancies [3]. Most cases arise from the muco-cutaneous junction [4]. AM behaves different from more common CM due to variation in biology, pathologic features, mutational profile and clinical profile. Patients usually present with bleeding per rectum, anal mass, anorectal pain or altered bowel habits [1,2]. *BRAF* mutation may be detected in about 50% patient with CM, are seen in only 4.5–15% patients in AM [5–9]. *Kit* mutations are detected at a higher frequency, 12–35% patients with AM compared with 2–10% patients with CM [7–12]. On histopathologic examination, pigmentation is seen only in about one half of cases [13,14].

AM are often misdiagnosed initially due to rarity, concealed anatomic location, amelanotic histology and pathological variations [15]. Most patients present at an advanced stage with regional lymph node involvement in approximately 60%, and distant metastases in approximately 30% [1,2]. Ballantine staging system is most commonly used as American Joint Committee on Cancer staging system does not include AM [16].

Clinical course of AM is aggressive with 5-year overall survival of 20% compared to 89% for cutaneous melanoma. It varies from 32% in localized disease, 16% in regional disease to 5% in metastatic disease [17].

Melanoma in very rare in Indian population and there is lack of literature regarding AM. The aim of our study was to analyze the clinicopathological profile and outcomes of patients with AM from a tertiary cancer care center in India.

## Methodology

This retrospective study was conducted at the Department of Medical Oncology from January 2016 to March 2020 after approval from institutional ethics review board. All patients with histologically proven anal, rectal or anorectal melanoma were included in the study. Baseline demographics profile, presenting symptoms, staging, histopathologic features, detailed treatment summary, response to treatment, pattern of relapse were recorded from case records. Date of diagnosis, date of relapse, time to relapse (defined as period from date of diagnosis to date of relapse) and overall survival (defined as period from date of diagnosis to death or last follow up) were also recorded.

The staging was carried out in accordance with the Ballantine staging system. Only the anal canal or rectum was involved in stage 1, pelvic lymph nodes were also involved in stage 2 and distant spread was identified in stage 3 [16].

### Statistical analysis

Data were analyzed by Stata 14 (TX, USA) and presented in mean ± standard deviation/median (range) and frequency (percentage). Categorical variables were compared by chi-squared or Fisher’s exact test. Continuous variables following normal distribution were compared by independent T test. Data not following normal distribution were compared by Wilcoxon rank sum test. Time to event was calculated by Kaplan–Meier analysis and survival compared by the log rank test. Univariate and multivariable cox regression analysis was used to find independent predictors of survival and mortality. Adjusted/unadjusted death or last follow up was used for calculating hazard ratio. A p-value < 0.05 was considered statistically significant.

## Results

### Baseline characteristics

Twenty-seven patients with AM were treated during above period. Baseline characteristics have been summarized in Table 1. Median age was 57 years (range: 26–80 years). Majority (70.4%; n = 19) were males. Median duration of symptoms before presenting to hospital was 4 months (range: 1–12 months). Common presenting symptoms were anorectal mass (70.4%; n = 19), bleeding per rectum (70.4%; n = 19) and perianal pain (66.6%; n = 18). Most common location of primary was anal canal (37%), followed by rectum (33.3%).

**Table 1.** Baseline characteristics of patients with anorectal melanoma.

Parameter	n = 27 (%)
Median age (years)	57 (26–80).
Male/female	19/8 (70.4% males)
Median symptom duration (months)	4 (1–12)
Presenting symptoms Anorectal mass Bleeding per rectum Perianal pain Constipation Weight loss Fatigue Diarrhea Loss of appetite Inguinal mass	19 (70.4%) 19 (70.4%) 18 (66.6%) 11 (40.7%) 8 (29.6%) 2 (7.4%) 2 (7.4%) 2 (7.4%) 1 (3.7%)
Location of primary tumor Anal canal Anorectal Rectal	10 (37.0%) 8 (29.6%) 9 (33.3%)
Ballantine staging Stage 1 Stage 2 Stage 3	6 (22.2%) 9 (33.3%) 12 (44.4%)
Depth of invasion Mucosa Submucosa Muscularis Not known	0 4 (14.8%) 18 (66.6%) 5 (18.5%)
Pigmentation Amelanotic Melanotic	15 (55.5%) 12 (44.4%)
Lymph node involvement	18 (66.6%)
Distant metastasis Liver Lung Bones	8 (29.6%) 6 (22.2%) 1 (3.7%)

On histopathology, most tumor were amelanotic (55.5%; n = 15) and muscularis layer was infiltrated in 66.6% (n = 18). Seven patients (25.9%) were misdiagnosed at initial presentation. Four patient were misdiagnosed on initial histopathology report from other centers (adenocarcinoma, sarcoma, neuroendocrine carcinoma and poorly differentiated carcinoma in one patient each), and three patients had clinical diagnosis of hemorrhoids and were operated inadvertently. *KIT* mutation testing and *BRAF* mutation testing was done in six (22.2%) and three (11.1%) patients, respectively. One patient each had exon 11 *KIT* mutation (16.6%) and *BRAF* mutation (33.3%). For staging MRI and positron emission tomography-computed tomography (PET-CT) was done in 11 (40.7%) patients, only PET-CT in six (22.2%) patients and contrast CT in ten (37.0%) patients. Most patients presented at an advanced stage (77.7% stage II and III). Lymph node involvement was present in 18 (66.6%) patients at diagnosis. Most common site of distant metastasis was liver (29.6%), followed by lung (22.2%).

### Management

Front-line surgical intervention was done in 13 (86.6%) out of 15 patients in stage I and II patients. Two patients refused surgery, one underwent chemoradiation and other patient opted for close follow up. Initial management of AM has been summarized in Table 2. Abdomino-perineal resection (APR) was performed in six (46.1%) patients, and wide local excision (WLE) in four (30.7%) patients. Three (23.0%) patient were clinically diagnosed as hemorrhoids and underwent local resection.

**Table 2.** First line of management in patients with anorectal melanoma.

Treatment modality	n = 27 (%)
First-line treatment Surgery Palliative chemotherapy Best supportive care Chemoradiation Observation	13 (48.1%) 9 (33.3%) 3(11.1%) 1 (3.7%) 1 (3.7%)
Initial surgery (n = 13) APR WLE Others^†^	6 (46.1%) 4 (30.8%) 3 (23.1%)
Adjuvant chemotherapy	1 (3.7%)
Adjuvant radiotherapy	2 (7.4%)
Palliative radiotherapy	6 (22.2%)
First-line palliative chemotherapy Dacarbazine Immunotherapy Taxane^‡^	5 (18.5%) 2 (7.4%) 2 (7.4%)

† Surgery done considering hemorrhoidectomy.

‡ Nab-paclitaxel and docetaxel in one patient each.

APR: Abdomino-perineal resection; WLE: Wide local excision.

Nine (75.0%) of 12 advanced-stage patients were started on upfront palliative therapy. Chemotherapy was administered in seven (77.7%) and immunotherapy in two (22.2%) patients. Most commonly administered chemotherapeutic was dacarbazine in five patients. Median number chemotherapy cycles administered were 3.

In patients receiving upfront palliative chemotherapy, response evaluation was done in six patients. Best response observed was partial remission (PR) in one (16.6%) patient, stable disease (SD) in two (33.3%) patients and progressive disease (PD) in three patients. Two patients received first-line palliative immunotherapy (one nivolumab and one pembrolizumab), partial response was achieved in one patient (50%) and progressive disease in other. Three patients also received immunotherapy in further lines, SD response was observed in one patient, progressive disease in one and evaluation could not be done in one patient. Six patients (22.2%) also received palliative radiotherapy. Three patients were advised best supportive care due to poor performance status at presentation.

Overall, five patients received immunotherapy, two in first line and three in second line. Out of four response evaluable patients, one (25.0%) patients achieved PR.

### Outcomes

**Figure 1.**
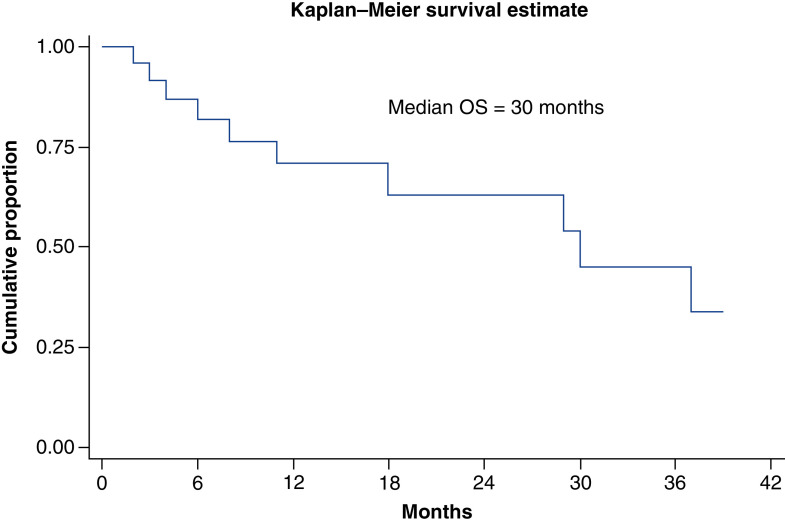
Kaplan–Meier curve showing overall survival in study population at median follow-up time of 11 months (range: 1–36 months). OS: Overall survival.

**Figure 2.**
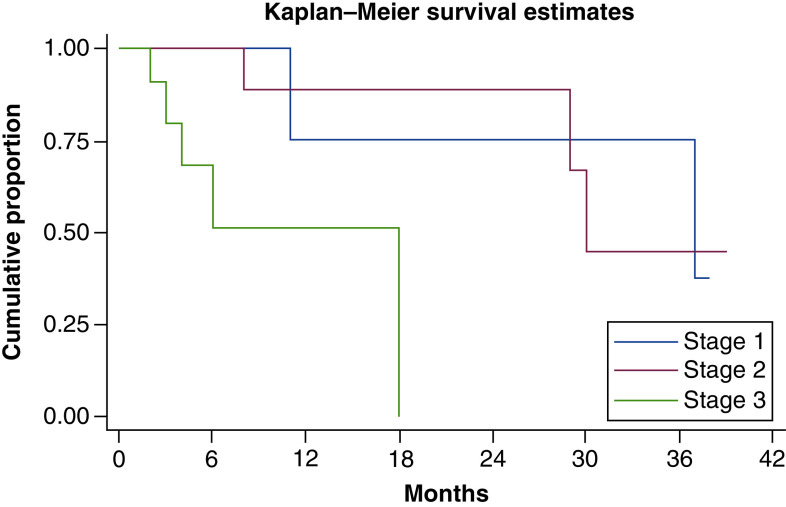
Kaplan–Meier curve showing overall survival with stage. Median overall survival was 37, 30 and 18 months in stage I, II and III, respectively.

At median follow-up time of 11 months, median overall survival was 30 months (11 months – not reached). Overall survival at 1 and 3 years was 70 and 45%, respectively. There was significant difference in survival between Ballantine stages. Median overall survival in stage I, stage II and stage III was 37, 30 and 18 months, respectively. Overall survival and survival in different stages has been shown in Figures 1 & 2. On univariate analysis, Ballantine stage 3 (p = 0.014), weight loss at presentation (p = 0.021) and presence of lung metastasis at diagnosis (p = 0.0007) were predictors of poor survival. On multivariate analysis, Ballantine stage 3 and weight loss at presentation were predictors of poor survival.

Patient with BRAF mutation: The patient is a 57-year-old female who presented with constipation and mass protruding from the anal canal. On evaluation, she was found to have Ballantine stage 2 anal melanoma (presence of pelvic lymph nodes) and she underwent APR with pelvic lymph node dissection. The postoperative specimen was positive for BRAF V600E mutation. She was kept on observation as the patient was unwilling for the adjuvant therapy. At a follow up of 15 months, the patient was disease-free.

Patient with cKIT mutation: The patient is a 34-year-old male, presented with bleeding per rectum, pain and mass per rectum. He underwent WLE only as he had Ballantine stage 1 rectal melanoma. He had a local relapse 13 months after surgery for which he underwent repeat WLE. After 7 months, he had a systemic relapse and was started on nivolumab every 3 weeks. He received four cycles of nivolumab and achieved PR. However, he progressed after eight cycles. Mutation analysis showed the presence of cKIT exon 11 mutations and was started on imatinib, but did not show any response to imatinib.

### Relapse

**Figure 3.**
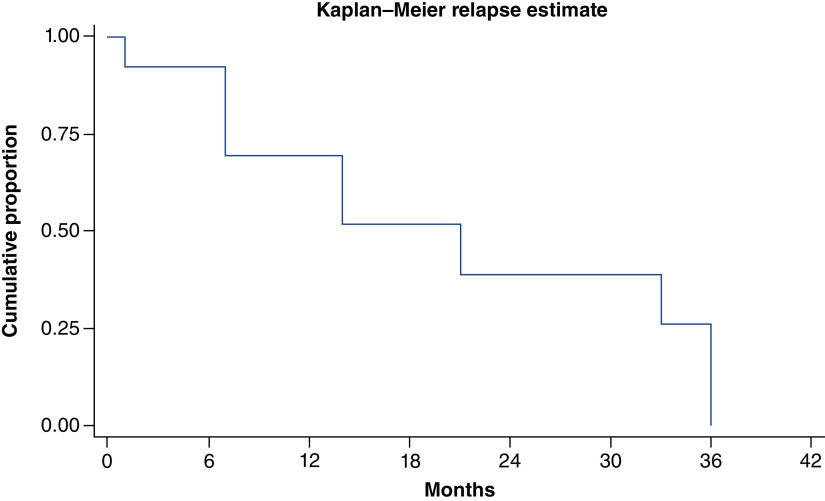
Kaplan–Meier curve showing probability of relapse following surgery in anorectal melanoma. Median time to relapse after surgery was 21 months (7 months – not reached).

**Figure 4.**
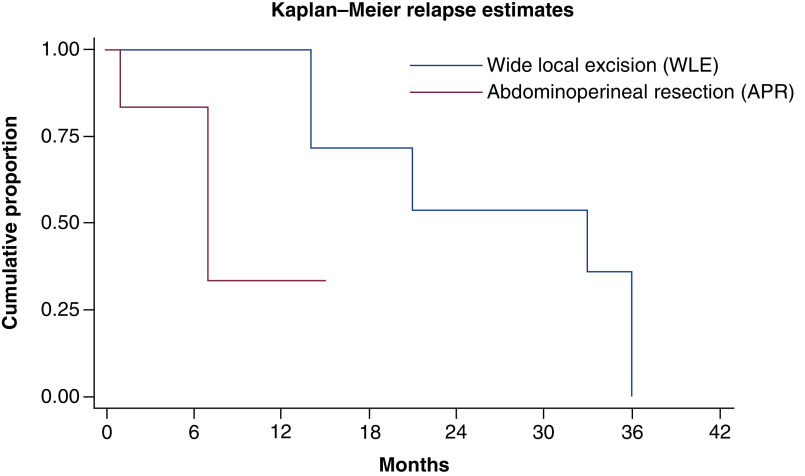
Kaplan–Meier curve showing probability of relapse following wide local excision and abdomino-perineal resection in anorectal melanoma. Median time to relapse was 33 months with WLE and 7 months with APR. APR: Abdomino-perineal resection; WLE: Wide local excision.

69.2% (nine out of 13) patients relapsed after initial surgery. Most patients (88.8%) had widespread disease at the time of relapse. One patient (11.1%) had relapse at local site only. Most common sites at relapse were lymph node, lung, liver and bones. Characteristics of patient at the time of relapse have been summarized in Table 3. At median follow-up time of 14 months, median time to relapse was 21 months (Figure 3). At time of relapse, one patient with localized disease underwent repeat surgery, four patients were started on palliative chemotherapy, two patients were kept on best supportive care due to poor performance status. One patient with kit mutation was started on imatinib 400 mg but had no response to therapy. On univariate analysis, type of initial surgery (APR) was only predictor of relapse. Figure 4 shows probability of relapse, following WLE and APR in AM.

**Table 3.** Characteristics and management at time of relapse in anorectal melanoma.

Parameter	n = 9 (%)
Median time to relapse	21 months (7 months – not reached)
Sites involved at time of relapse Local^†^ Lymph nodes Lung Liver Bones Peritoneal/omental Others (pancreas, spleen, brain, urinary bladder)	7 (77.7%) 6 (66.6%) 6 (66.6%) 5 (55.5%) 4 (44.4%) 2 (22.2%) 1 (11.1%)
Treatment at relapse Palliative chemotherapy Observation WBRT Surgery Best supportive care	4 (44.4%) 2 (22.2%) 2 (22.2%) 1 (11.1%) 1 (11.1%)

† Local only relapse in one patient.

WBRT: Whole brain radiotherapy.

## Discussion

Patients in our study were slightly younger with a median age of 57 years and male preponderance with male-to-female ratio of 2.4:1. Previous studies from India also showed younger age at presentation compared with western population with mean age ranging from 49 to 53 years [18–20]. Most common location was anal or anorectal in two third of patient, which is similar to reported literature [21,22]. Majority (77.7%) patients in our study had either regional (stage II) or disseminated disease (stage III) at the time of initial diagnosis. Previous studies of AM from India reported higher proportion of patients with stage II and III disease (82 and 93%, respectively) [19,20]. However, western data showed slight greater proportion of local (stage I) disease [17,23]. Involvement of regional lymph nodes at presentation was observed in 70% patients in our study, which is greater than in previous studies (20–60% regional lymph node involvement) [1,24]. This may be due to delayed presentation at healthcare facilities due to nonspecific symptoms, amelanotic histology and misdiagnosis [25].

Fifty-five percent tumors were amelanotic in our study, which is similar to the previous ones. Amelanotic tumors may have poorer prognosis because they are more invasive and have more chances of misdiagnosis [13,14]. Diagnosis may be delayed due to wrong clinical diagnosis or pathological reporting and varying histological findings. Many cases may be initially reported as adenocarcinoma, lymphoma, sarcoma or hemorrhoids [26,27].

Surgery is mainstay of management in early-stage AM. However, no surgical modality has been proven to be superior to other as retrospective studies have not shown any benefit of APR over WLE. In our study, WLE was performed in 53.8% patients and APR in rest. There was no difference in overall survival, relapse rate between these two groups, however, local relapses were more frequent in patients undergoing WLE [28,29].

There is scarcity of literature on the role of chemotherapy in AM. In our study, seven patients received palliative chemotherapy in first line and five patients in further lines. Best response was partial response in 16.6% patients and SD 33.3% patients who received palliative chemotherapy in first line; no responses were observed in further lines of chemotherapy.

Data on immunotherapy in AM are lacking, and the response rates are low compared with the cutaneous melanoma. In our study, total five patients received single-agent immunotherapy, two in first-line and three in second-line metastatic setting. PR was observed in 25% of evaluable patients. Previous studies have shown responses in about 23% patients receiving single-agent nivolumab and 37% in patient on nivolumab and ipilimumab combination [30]. In another study were noted in 22% of previously untreated patients [31]. However, these studies are post hoc analysis and have small number of patients.

In our study, median survival in stage I, II and III AM was better than the previous studies [32]. This may be explained by improved surgical techniques and advent of newer therapies over the last two decades. Ballantine stage 3 and weight loss were predictors of poor survival in our study. Disease stage has been consistently shown to be prognostic for overall survival in the previous studies. Other important prognostic factors include lymph nodal involvement and depth of tumor [4,21,22,29].

Our study has some limitations due to small-sample size, short follow up and retrospective nature. *BRAF* and *KIT* mutation status could be performed in few patients only. Only few patients could receive immunotherapy due to economic reasons.

## Conclusion

AM presents at an advanced stage and have poor outcomes. Pathological features and mutational profile differs from more common cutaneous melanomas. Patients are often misdiagnosed due to location and pathological variations. Stage at diagnosis is most important prognostic factor. Surgery is standard treatment for early-stage disease; however, optimal management in adjuvant and metastatic setting is unclear. Larger prospective studies are required to address the management of this entity and define the role of targeted and immunotherapy.

## Future perspective

There is lack of understanding of tumor biology and molecular signatures in AM. Due to rarity of condition, there is lack of prospective randomized trials and thus management strategy is not well defined. The role of PET-CT in staging workup remains to be explored. The role of adjuvant therapy, targeted therapy and immunotherapy remains poorly defined. All patients should be encouraged to participate in prospective collaborative studies to identify the oncogenic driver mutations, and establish role of available therapeutic modalities.

**Summary points**

- Anorectal melanoma (AM) is a rare subset, which constitutes about 1.3% of all melanomas.
- Median age at diagnosis was 57 years with a male-to-female ratio of 2.4:1.
- Most common location was anal or anorectal in about two third of patient.
- Majority patients had either regional (stage II) or disseminated disease (stage III) at the time of initial diagnosis.
- More than half of tumors were amelanotic on histopathology.
- Involvement of regional lymph nodes at presentation was observed in 70% patients in our study, which way be attributed to delayed presentation, amelanotic histology and misdiagnosis.
- Diagnosis may be delayed due to wrong clinical diagnosis or pathological reporting and varying histological findings. Many cases may be initially reported as adenocarcinoma, lymphoma, sarcoma or hemorrhoids.
- Surgery is mainstay of management in early-stage AM. No surgical modality (wide local excision or abdomino-perineal resection) has been proven to be superior to other; however, local relapses were more frequent in patients undergoing wide local excision.
- Best response to chemotherapy was partial response in 16.6% patients and stable disease 33.3% patients who received palliative chemotherapy in first line; no responses were observed in further lines of chemotherapy.
- Data on immunotherapy in AM are lacking, and the response rates are low compared with the cutaneous melanoma. In our study in five patients receiving immunotherapy, partial remission was observed in 25% of evaluable patients.
- Ballantine stage 3 and weight loss were predictors of poor survival in our study. Disease stage has been consistently shown to be prognostic for overall survival in the previous studies.
